# Prospective cohort study assessing the efficacy of dermoscopy-guided combination therapy using CO_2_ and 1064-nm lasers for junctional nevus

**DOI:** 10.3389/fmed.2026.1708646

**Published:** 2026-02-09

**Authors:** Yun Wang, Sainan Zhang

**Affiliations:** Department of Dermatology, The Affiliated Huai'an Hospital of Xuzhou Medical University, The Second People's Hospital of Huai'an, Huai'an, China

**Keywords:** 1064-nm laser, CO_2_ laser, dermoscopy, junctional nevus, prospective cohort study

## Abstract

**Background:**

Junctional nevus is a common benign cutaneous tumor with a potential risk of malignant transformation. Traditional CO_2_ laser treatment often leads to residual lesions and recurrence due to limited vaporization depth. Dermoscopy enables real-time visualization of lesions, while the 1064-nm laser specifically targets melanin. This study evaluates the efficacy and safety of dermoscopy-assisted CO_2_ laser therapy combined with 1064 nm laser treatment for junctional nevus.

**Methods:**

From January 2023 to October 2024, 153 patients diagnosed with junctional nevus were recruited and allocated into an experimental group (*n* = 77) and a control group (*n* = 76). The control group received CO_2_ laser treatment guided by naked-eye observation, while the experimental group underwent dermoscopy-assisted CO_2_ laser therapy combined with 1064 nm laser treatment. Clinical efficacy, wound healing, pain levels, scar formation, patient satisfaction, and recurrence rates were assessed during a 6-month follow-up.

**Results:**

The total effective rate in the experimental group was 96.10%, which was significantly higher than the 86.84% observed in the control group (χ^2^ = 4.220, *P* = 0.040). Vancouver Scar Scale (VSS) scores in the experimental group were significantly lower than those in the control group at both 1 week (6.47 ± 1.08 vs. 7.82 ± 1.02; *t* = 7.947, *P* < 0.001) and 1 month (3.02 ± 0.39 vs. 3.81 ± 0.48; *t* = 11.18, *P* < 0.001) post-treatment. Patient satisfaction with aesthetics (96.10%) and treatment efficacy (94.80%) was higher in the experimental group compared to the control group (aesthetics: 86.84%, χ^2^ = 4.220, *P* = 0.040; treatment efficacy: 84.21%, χ^2^ = 4.585, *P* = 0.032), whereas satisfaction with comfort did not differ significantly. Recurrence rates at 2 months (2.60%) and 6 months (7.80%) post-treatment were significantly lower in the experimental group than in the control group (2 months: 10.53%, χ^2^ = 3.936, *P* = 0.047; 6 months: 22.37%, χ^2^ = 6.362, *P* = 0.012).

**Conclusion:**

The combination of dermoscopy-assisted CO_2_ laser and 1064 nm laser therapy for the treatment of junctional nevus enhances therapeutic efficacy, diminishes scar severity, increases patient satisfaction, and reduces recurrence rates.

## 1 Introduction

Junctional nevus is a prevalent benign cutaneous tumor characterized by clusters of nevus cells situated at the epidermal-dermal junction. It commonly manifests on the extremities and transitional epithelial regions, particularly among adolescents, and is frequently misdiagnosed as melanoma (1, 2). Although junctional nevus are typically benign, the active proliferation of nevus cells presents a potential risk for malignant transformation into dysplastic nevus. Conventional surgical excision often results in recurrence, necessitating careful consideration in the clinical diagnosis and management of junctional nevus (3, 4). Given the increasing demand for favorable cosmetic outcomes, traditional interventions such as surgical excision are limited by the risk of conspicuous scarring. Laser therapy, characterized by its minimally invasive approach and rapid recovery, has emerged as the preferred treatment modality for cutaneous lesions (5). Nevertheless, the commonly employed CO_2_ laser, despite its efficacy in removing nevus cells, has a restricted vaporization depth and is unable to completely eradicate nevus cells located in the deeper dermis, frequently leading to residual lesions and recurrence. Furthermore, it is challenging to ascertain with the naked eye whether nevus cells have been entirely removed during treatment. Repeated interventions not only elevate the risk of recurrence but may also promote malignant transformation of nevus cells.

In recent years, dermoscopy has been extensively utilized as a non-invasive, real-time, dynamic microscopic imaging modality for the diagnosis and treatment monitoring of skin lesions (6, 7). This technique offers detailed visualization of skin structures, eliminates the need for biopsy-related trauma, involves no radiation exposure, and is generally well accepted by patients. The 1064 nm laser specifically targets melanin, rendering it an effective option for the treatment of pigmented disorders. Theoretically, the combined application of CO_2_ and 1064 nm lasers may synergistically enhance therapeutic outcomes by leveraging their respective advantages. Accordingly, this study aims to evaluate the clinical efficacy of dermoscopy-assisted combined CO_2_ and 1064 nm laser therapy for junctional nevus through a prospective cohort study, with the objective of informing optimized clinical treatment strategies.

## 2 Methods

### 2.1 Study design

This prospective cohort study aimed to assess the clinical efficacy of dermoscopy-assisted combined CO_2_ and 1064 nm laser treatment for junctional nevus. Conducted from January 2023 to October 2024, the study enrolled 153 patients who were allocated into control and experimental groups, each subjected to distinct treatment protocols. A follow-up period of 6 months was implemented to evaluate outcomes.

### 2.2 Study subjects

The study comprised 153 patients diagnosed with junctional nevus who attended the Dermatology and Venereology Departments of Huai'an Hospital Affiliated with Xuzhou Medical University (Huai'an Second People's Hospital), the Dermatology Department of Huai'an 82nd Hospital, the Dermatology Department of Jiawang District People's Hospital in Xuzhou, and the Dermatology Department of the Affiliated Hospital of Xuzhou Medical University between January 2023 and October 2024.

**Inclusion criteria:** (1) age between 12 and 60 years; (2) clinical and dermoscopic diagnosis of junctional nevus; and (3) voluntary participation with the ability to attend scheduled follow-up visits.

**Exclusion criteria:** (1) patients predisposed to keloid formation; (2) patients presenting with other benign skin tumors, such as compound nevus or seborrheic keratosis; (3) individuals with coagulation disorders; (4) presence of active infection at the treatment site; (5) pregnant or breastfeeding women; and (6) patients with severe chronic diseases that impair wound healing, including diabetes mellitus.

### 2.3 Research methods

#### 2.3.1 Equipment

The study utilized a fractional KL-type super-pulsed CO_2_ laser (Jilin Keying Laser Co., Ltd.), a Q-switched Nd:YAG laser operating at 1064 nm [KL-M(H) model, Jilin Keying Laser Co., Ltd.], a handheld confocal laser scanning microscope (Vivascopel500, Lucid Inc., USA), and a non-invasive real-time pathological analysis system (Kang'ao Technology Group Co., Ltd.) equipped with an 830 nm laser source, adjustable output power ranging from 0 to 16.0 mW, 30 × objective magnification, and a 5 μm axial scanning interval. Additionally, a dermoscope (Dermoscopy-IIL, Beijing Demate Jiekang Technology Development Co., Ltd.) was employed.

#### 2.3.2 Treatment protocol

The control group underwent CO_2_ laser treatment guided solely by naked-eye observation. Following routine disinfection of the junctional nevus area, CO_2_ laser ablation was performed until no visible pigmentation remained, which was defined as the treatment endpoint. In contrast, the experimental group received a combined treatment involving CO_2_ and 1064 nm lasers, assisted by dermoscopy. After routine disinfection, CO_2_ laser ablation was initially applied until no pigmentation was visible to the naked eye. Subsequently, dermoscopy was employed to monitor the treated area. To ensure accurate assessment despite potential laser-induced inflammation, the following criteria were applied: the treatment area was gently cleaned with normal saline, and the dermoscopy was gently touched to the skin. The main criterion for identifying residual lesions is the persistent presence of pigment networks, which is a characteristic of junctional nevi and distinguishes them from the surrounding diffuse erythema or coagulation whiteness. Any such residual pigmentation identified was eliminated using the 1064 nm laser until no residual pigment network was detectable under dermoscopy, thereby establishing the treatment endpoint. Postoperatively, bovine basic fibroblast growth factor gel and fusidic acid ointment were applied topically for 7 days, accompanied by recommendations for wound protection and sun avoidance. The CO_2_ laser parameters: forced pulse 0.3–2.0 W; 1064 nm laser parameters: spot size 3–4 mm, energy density 2.5–3.0 J/cm^2^.

### 2.4 Outcome measures

#### 2.4.1 Clinical efficacy

Two months following treatment, clinical efficacy was assessed by evaluating the removal of junctional nevus.

- Markedly effective: no pigment residue remains; the skin surface appears smooth and even.
- Effective: no or minimal pigment deposition observed; minor scarring is evident.
- Ineffective: marked pigment deposition and prominent scarring are evident.

The total effective rate is calculated as the sum of markedly effective cases and effective cases divided by the total number of cases, multiplied by 100%.

#### 2.4.2 Wound healing

Wound healing was evaluated based on the time to complete epithelialization, the duration of scab presence, and the persistence of erythema following treatment.

- Wound healing time is defined as the number of days from the completion of treatment to the achievement of complete epithelialization.
- Scab duration: the number of days from the completion of treatment until the natural shedding of the scab.
- Duration of erythema: the number of days from the completion of treatment until the complete resolution of redness.

#### 2.4.3 Pain assessment

Pain was assessed immediately after treatment and again 24 h post-treatment using the Visual Analog Scale (VAS) (8). Participants rated their pain on a scale from 0 to 10, where 0 represents no pain and 10 denotes the most severe, unbearable pain.

#### 2.4.4 Scar assessment

Scar severity was evaluated at 2 week and 1 month following treatment using the Vancouver Scar Scale (VSS) (9). This scale assesses scar characteristics including color (0–3 points), vascularity (0–3 points), thickness (0–4 points), and pliability (0–5 points). Lower total scores correspond to less severe scarring, indicating skin that more closely resembles normal tissue.

#### 2.4.5 Patient satisfaction

Two months following treatment, patients completed a questionnaire assessing their satisfaction with appearance, treatment efficacy, and comfort using a 0–10 scale. Scores of 8 or higher were classified as satisfactory. The satisfaction rate was calculated as the number of satisfied cases divided by the total number of cases, multiplied by 100%.

#### 2.4.6 Recurrence

Recurrence was documented at 2- and 6-months following treatment and was defined as the reappearance of pigmented nevus at or near the original treatment site. The recurrence rate was calculated as the number of recurrent cases divided by the total number of cases, multiplied by 100%.

### 2.5 Statistical analysis

Data were analyzed using SPSS version 26.0. Continuous variables were presented as mean ± standard deviation (SD) and compared between groups using the independent samples *t*-test. Categorical variables were expressed as percentages (%) and compared using the chi-square test. A *p*-value of less than 0.05 was considered statistically significant.

## 3 Results

### 3.1 Baseline characteristics

The control group comprised 35 males and 41 females, aged between 13 and 60 years, with a mean age of 36.78 ± 10.78 years. The number of lesions per participant ranged from 1 to 10, with a mean of 3.65 ± 1.28. The experimental group consisted of 36 males and 41 females, aged between 12 and 59 years, with a mean age of 36.28 ± 10.84 years. The number of lesions ranged from 1 to 11, averaging 3.58 ± 1.32. No statistically significant differences were observed in baseline characteristics between the two groups (Table 1).

**Table 1:** Basic characteristics of the study population.

Variable		**Control group**	**Experimental group**	***t/χ^2^***	***P***
Gender	Male [n (%)]	35 (46.05)	36 (46.75)	0.075	0.931
	Female [n (%)]	41 (53.95)	41 (53.25)		
Age (years)		36.78 ± 10.78	36.28 ± 10.84	0.286	0.775
Lesions (numbers)		3.65 ± 1.28	3.58 ± 1.32	0.339	0.74

### 3.2 Comparative analysis of clinical efficacy

**Figure 1:**
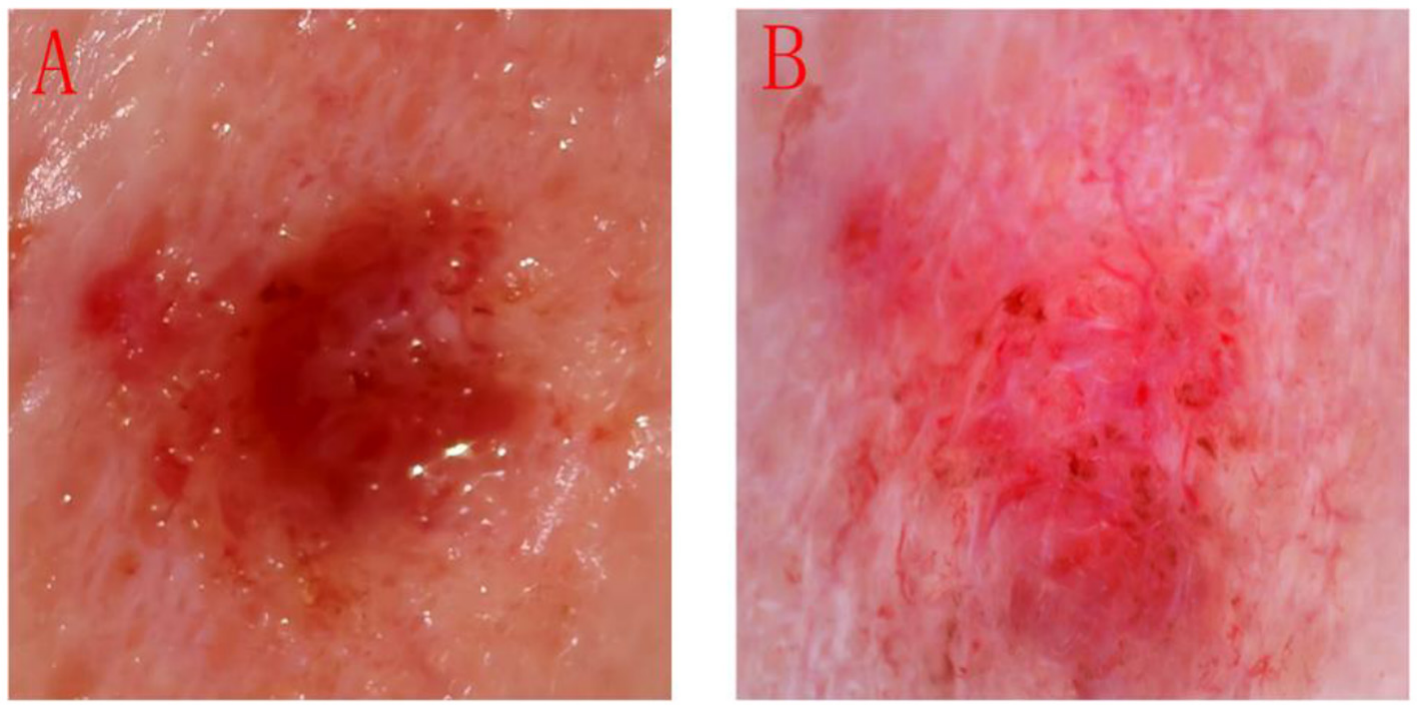
Image of the patient in the experimental group needed 1064 nm laser treatment after undergoing CO_2_ laser treatment. **(A)** Observation by naked-eye; **(B)** Observation by dermoscopy.

**Figure 2:**
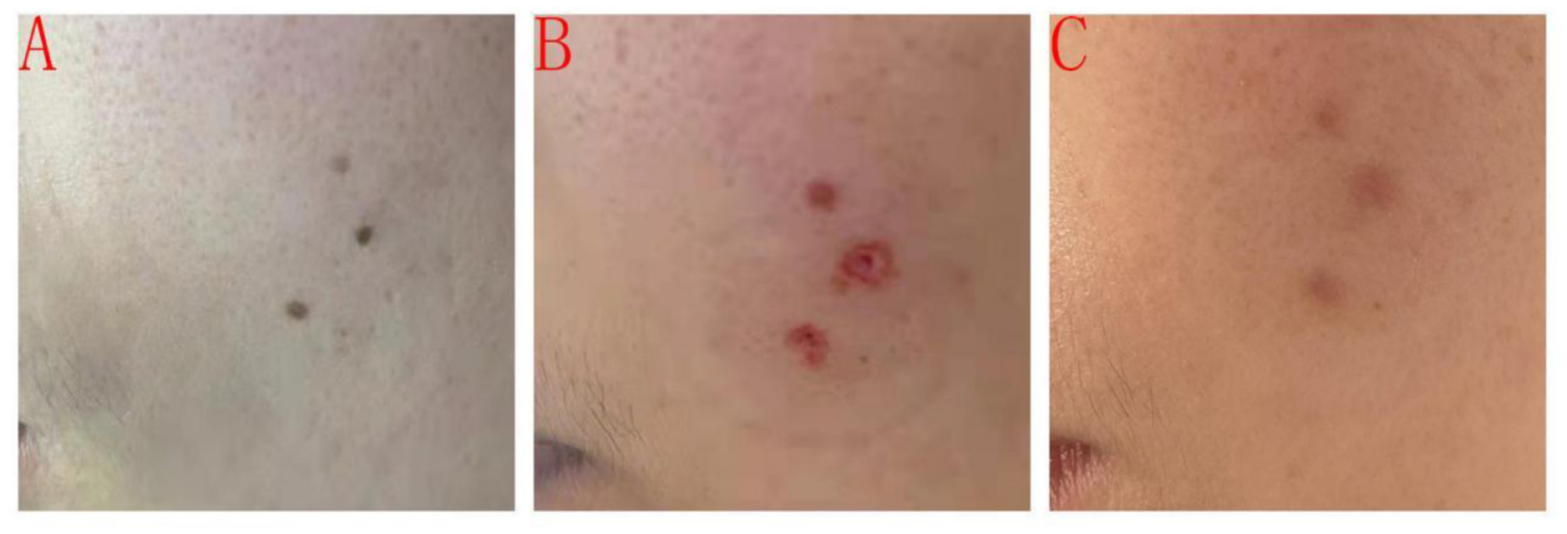
Typical case in the experimental group before and after treatment. **(A)** Patient's facial border nevus before treatment; **(B)** Immediate facial condition after combined CO_2_ laser and 1064-nm laser treatment; and **(C)** Facial condition 2 months after treatment.

Following treatment, the total effective rate in the experimental group (96.10%) was significantly greater than that observed in the control group (86.84%; *P* < 0.05), as presented in Table 2. The image of the patient in the experimental group needed 1064 nm laser treatment after undergoing CO_2_ laser treatment is shown in Figure 1, and an illustrative case from the experimental group is depicted in Figure 2.

**Table 2:** Comparison of clinical efficacy between two groups [n (%)].

**Group**	***n***	**Markedly effective**	**Effective**	**Ineffective**	**Total effective**
Experimental	77	28 (36.36)	46 (59.74)	3 (3.90)	74 (96.10)
Control	76	25 (32.89)	41 (53.95)	10 (13.16)	66 (86.84)
*χ2*		4.220			
*P*		0.040			

### 3.3 Comparative analysis of wound healing

No statistically significant differences were observed between the groups regarding wound healing time, scab duration, or erythema duration (*P* > 0.05), as presented in Table 3.

**Table 3:** Comparison of wound healing conditions between two groups (mean ± SD).

**Group**	***n***	**Wound healing time (days)**	**Scab formation time (days)**	**Duration of erythema (months)**
Experimental	77	8.211 ± 0.63	2.830 ± 0.41	2.970 ± 0.42
Control	76	8.371 ± 0.54	2.800 ± 0.39	3.040 ± 0.58
*t*		0.624	0.464	0.854
*P*		0.534	0.644	0.395

### 3.4 Comparison of pain and scarring

VAS scores for pain assessed immediately post-treatment and at 24 h demonstrated no significant differences between the experimental and control groups (*P* > 0.05). In contrast, VSS scores for scar evaluation at 1 week and 1 month post-treatment were significantly lower in the experimental group compared to the control group (*P* < 0.05), as presented in Table 4.

**Table 4:** Comparison of pain and scar conditions between two groups (mean ± SD).

**Group**	***n***	VAS scores		VSS scores	
		**Post-operation (0 h)**	**Post-operation (24 h)**	**Post-operation (1 week)**	**Post-operation (1 month)**
Experimental	77	4.730 ± 0.51	1.020 ± 0.17	6.471 ± 0.08	3.020 ± 0.39
Control	76	4.580 ± 0.62	1.010 ± 0.24	7.821 ± 0.02	3.810 ± 0.48
*t*		1.635	0.766	7.947	11.18
*P*		0.104	0.298	< 0.001	< 0.001

### 3.5 Comparison of patient satisfaction levels

The experimental group demonstrated significantly greater satisfaction with both appearance and treatment efficacy compared to the control group (*P* < 0.05). However, no significant difference was observed between the groups regarding comfort satisfaction (*P* > 0.05), as presented in Table 5.

**Table 5:** Comparison of satisfaction between two groups [n (%)].

**Group**	***n***	**Appearance**	**Treatment effect**	**Comfort**
Experimental	77	74 (96.10)	73 (94.80)	69 (89.61)
Control	76	66 (86.84)	64 (84.21)	69 (90.79)
*χ^2^*		4.220	4.585	0.060
*P*		0.040	0.032	0.806

### 3.6 Comparison of recurrence rates

The recurrence rates at 2 and 6 months following treatment were significantly lower in the experimental group compared to the control group (*P* < 0.05), as presented in Table 6.

**Table 6:** Comparison of recurrence between two groups [n (%)].

**Group**	***n***	**Post-operation (2 months)**	**Post-operation (6 months)**
Experimental	77	2 (96.10)	6 (94.80)
Control	76	8 (86.84)	17 (84.21)
*χ^2^*		3.936	6.362
*P*		0.047	0.012

## 4 Discussion

The differential diagnosis between melanoma and junctional nevus is critical in clinical practice. Reflectance confocal microscopy (RCM) morphological classification of junctional nevus predominantly reveals a ring-like pattern, which corresponds to the proliferation of melanocytes at the single-cell level (4). Furthermore, studies have demonstrated that four-color fluorescence *in situ* hybridization effectively distinguishes acral melanoma from benign acral junctional nevus (10). Additionally, deep learning-based dermoscopy techniques can differentiate among various types of pigmented nevus, and image recognition algorithms contribute to enhancing diagnostic accuracy.

Junctional nevus is a common benign cutaneous tumor that carries a potential risk of malignant transformation, particularly in adolescents and in specific anatomical locations such as the extremities. The primary objectives of treatment include complete lesion removal, optimal cosmetic outcomes, and minimization of recurrence. In response to increasing cosmetic demands, minimally invasive laser therapies that facilitate rapid recovery have become the preferred treatment modalities. Although the CO_2_ laser is effective in ablating nevus cells, its limited vaporization depth poses challenges in achieving complete eradication of deep dermal nevus cells, often resulting in residual lesions and subsequent recurrence (11, 12). The 1064 nm laser, which selectively targets melanin, theoretically complements the CO_2_ laser by enhancing overall treatment efficacy through combined application (13, 14). Following CO_2_ laser ablation of the epidermal and superficial dermal pigment, dermoscopic examination can identify residual fine pigment networks, thereby guiding precise, targeted supplementary treatment with the 1064 nm laser. The high selective absorption of the 1064 nm laser by melanin enables effective destruction of deep nevus cells while minimizing collateral damage to surrounding normal tissue, thereby facilitating thorough and visually guided lesion clearance (15).

This prospective cohort study assessed the clinical efficacy of dermoscopy-assisted combined CO_2_ and 1064 nm laser treatment for junctional nevus. The results demonstrated that the experimental group exhibited a significantly higher overall effective rate and a lower recurrence rate compared to the control group, suggesting that dermoscopy-assisted combined treatment provides substantial benefits in enhancing treatment efficacy and minimizing recurrence.

Dermoscopy, a non-invasive, real-time, dynamic microscopic imaging modality, is widely accepted by patients and effectively mitigates the trauma associated with biopsy procedures. It provides detailed microscopic visualization of skin structures, enabling clinicians to more accurately identify residual nevus cells during treatment. This facilitates dynamic monitoring and precise determination of optimal treatment endpoints during laser therapy (16). The combined therapeutic approach significantly enhances efficacy, likely because the traditional CO_2_ laser, although effective in vaporizing epidermal and superficial dermal pigment, lacks the capacity to precisely detect deep residual nevus cell nests with the naked eye, particularly when pigment is obscured by thermal coagulation or carbonization (17). Dermoscopy's ability to penetrate the epidermis and deliver high-resolution imaging permits real-time, clear visualization of the morphology and depth distribution of nevus cell nests during treatment. Following CO_2_ laser ablation of visible pigment, dermoscopy enables the identification of residual fine pigment networks, thereby guiding targeted supplementary treatment with a 1064 nm laser. The 1064 nm wavelength exhibits high selective absorption by melanin, effectively destroying deep nevus cells while minimizing damage to surrounding normal tissue. This approach achieves thorough, visually guided lesion clearance, ultimately improving treatment efficacy and reducing recurrence rates.

It is worth noting that there is inherent uncertainty in the clinical and dermoscopy diagnosis of junctional nevi. We cannot completely rule out the possibility that a small number of these lesions may change in the future. However, precisely because of this uncertainty, it is particularly important to seek a strategy that can achieve a more thorough removal during treatment. The dermoscopy guided combined laser treatment method used in this study achieves deeper and more precise removal of pigment cells, not only aiming to reduce the recurrence rate of benign nevi, but also potentially minimizing the risk of residual potential atypical cells due to incomplete treatment.

Scar formation represents a significant concern in laser therapy, primarily attributable to excessive thermal injury that leads to destruction of the dermal matrix and aberrant tissue repair (18). Exclusive reliance on CO_2_ laser for complete removal of nevus cells often necessitates deeper treatment, resulting in unnecessary injury to the deep dermis. Furthermore, accurately determining the complete eradication of nevus cells during the procedure is challenging; repeated interventions increase the risk of recurrence and may potentially induce malignant transformation. In the present study, the experimental group initially employed CO_2_ laser to excise the primary lesion, followed by application of a 1064 nm laser, utilizing its selective photothermal effect to target residual deep pigment identified via dermoscopy. This combined approach mitigated excessive deep vaporization associated with CO_2_ laser use, thereby preserving the collagen architecture of the reticular dermis to the greatest extent possible. The findings demonstrated no significant differences between groups in wound healing time, scab duration, or erythema period; however, the experimental group exhibited significantly lower Vancouver Scar Scale (VSS) scores at 1 week and 1 month post-treatment compared to the control group. These results indicate that the 1064 nm laser's photomechanical or selective photothermal effects on melanin targets induce substantially less thermal damage to surrounding collagen than the extensive vaporization caused by CO_2_ laser ablation. Consequently, this approach reduces dermal structural damage, facilitates superior quality healing, and enhances patient satisfaction regarding aesthetic outcomes and overall treatment efficacy.

Patient satisfaction serves as a critical measure of treatment success. In this study, the experimental group demonstrated significantly greater satisfaction regarding both appearance and treatment outcomes compared to the control group. This finding suggests that dermoscopy-assisted combined treatment not only enhances therapeutic efficacy but also substantially improves cosmetic satisfaction. Furthermore, no significant difference was observed between the groups in terms of comfort satisfaction, indicating that the combined treatment enhances efficacy without exacerbating patient discomfort.

Although the dermoscopy-assisted combined CO_2_ and 1064 nm laser treatment used in this study may be higher than the single laser treatment in terms of cost and time, it achieves higher single-treatment efficiency, lower recurrence rate and better cosmetic outcome through precise guidance and complementary advantages. This potentially reduces the need for repeated treatments and the long-term costs associated with scar revision or managing recurrence (15), suggesting favorable cost-effectiveness over the complete treatment cycle. Furthermore, considering the resource limitations of small treatment centers, this plan should be prioritized for implementation in specialized treatment centers and mainly used for pigmented lesions that are clinically suspected to have a higher risk of malignant transformation.

## 5 Conclusion

In summary, dermoscopy-assisted combined CO_2_ and 1064 nm laser treatment for junctional nevus offers significant benefits, including enhanced treatment efficacy, reduced scar formation, increased patient satisfaction, and decreased recurrence rates, thereby representing a superior clinical option for managing junctional nevus. Nonetheless, this study is limited by a relatively small sample size and a short follow-up period. Future research should involve larger cohorts and extended follow-up durations to further substantiate the long-term efficacy and safety of this treatment protocol.

## Data Availability

The raw data supporting the conclusions of this article will be made available by the authors, without undue reservation.
